# The European MultiPartner IPF registry (EMPIRE): validating long-term prognostic factors in idiopathic pulmonary fibrosis

**DOI:** 10.1186/s12931-019-1271-z

**Published:** 2020-01-08

**Authors:** Tanja Tran, Martina Šterclová, Nesrin Mogulkoc, Katarzyna Lewandowska, Veronika Müller, Marta Hájková, Mordechai R. Kramer, Dragana Jovanović, Jasna Tekavec-Trkanjec, Michael Studnicka, Natalia Stoeva, Karel Hejduk, Ladislav Dušek, Samy Suissa, Martina Vašáková, for the EMPIRE registry, Beata Zolnowska, Vladimír Bartoš, Hradec Králové, Robert Slivka, Ladislav Lacina, Martina Doubková, Radka Bittenglová, Magdalena Martusewicz-Boros, Monika Žurková, Imrich Jonner, Amelia Szymanowska-Narloch, Małgorzata Sobiecka, Vladimíra Lošťáková, Marzena Trzaska-Sobczak, Richard Tyl, Pawel Sliwinski, Miklós Zsiray, Anikó Bohács, Sebastian Majewski, Hana Šuldová, Katarzyna Lewandowska, Bohumil Matula, Lenka Šišková, Ján Plutinský, Ana Jakić, Štefan Tóth, Zoltán Balikó, Margita Bučeková, Jana Pšíkalová, Tomasz Stachura, František Petřík, Jan Anton, Jaroslav Lněnička, Marina Roksandić Milenkovic, Imre Lajkó, Vladimír Řihák, Zsuzsanna Szalai, Paulina Jurek, Aleksander Kania, Štefan Laššán, Tatjana Pejcic, Pavel Reiterer, Lukasz Borucki, Renata Králová, Pavlína Musilová, Tomáš Snížek, Daniel Doležal, Jiří Homolka, Hana Hortvíková, Suzana Mladinov, Peter Palúch, Roman Hrdina, Maria Szilasi, Violeta Vučinić-Mihailović, Róbert Vyšehradský, Radka Mokošová, Agata Nowicka, Tatjana Radjenovic Petkovic

**Affiliations:** 1 Department of Epidemiology, Biostatistics, and Occupational Health, McGill University, Montreal, Quebec, Canada; 2 Centre for Clinical Epidemiology, Lady Davis Institute for Medical Research, Jewish General Hospital, Montreal, Quebec, Canada; 3 Department of Respiratory Medicine of the First Faculty of Medicine Charles University, Thomayer Hospital, Vídeňská 800, 140 59 Prague 4, Czech Republic; 4 Department of Chest Diseases, Faculty of Medicine, Ege University, Izmir, Turkey; 5 1st Department of Pulmonary Diseases, Institute of Tuberculosis and Lung Diseases, Warsaw, Poland; 6 Department of Pulmonology, Faculty of Medicine, Semmelweis University, Budapest, Hungary; 7 Clinic of Pneumology and Phthisiology, University Hospital Bratislava, Bratislava, Slovakia; 8 Institute of Pulmonary Medicine, Rabin Medical Center, Petah Tikva, Israel; 9 University Hospital of Pulmonology, Clinical Center of Serbia, Belgrade, Serbia; 10 Department of Pulmonology, Clinical Hospital Dubrava, Zagreb, Croatia; 11 Clinical Research Center Salzburg, Salzburg, Austria; 12 Department of Pulmonology, Acibadem City Clinic Tokuda Hospital, Sofia, Bulgaria; 13 Institute of Biostatistics and Analyses, Faculty of Medicine, Masaryk University, Brno, Czech Republic

## Abstract

**Background:**

Several registries of idiopathic pulmonary fibrosis (IPF) have been established to better understand its natural history, though their size and duration of follow-up are limited. Here, we describe the large European MultiPartner IPF Registry (EMPIRE) and validate predictors of long-term survival in IPF.

**Methods:**

The multinational prospective EMPIRE registry enrolled IPF patients from 48 sites in 10 Central and Eastern European countries since 2014. Survival from IPF diagnosis until death was estimated, accounting for left-truncation. The Cox proportional hazards regression model was used to estimate adjusted hazard ratios (HR) of death for prognostic factors, using restricted cubic splines to fit continuous factors.

**Results:**

The cohort included 1620 patients (mean age at diagnosis 67.6 years, 71% male, 63% smoking history), including 75% enrolled within 6 months of diagnosis. Median survival was 4.5 years, with 45% surviving 5 years post-diagnosis. Compared with GAP stage I, mortality was higher with GAP stages II (HR 2.9; 95% CI: 2.3–3.7) and III (HR 4.0; 95% CI: 2.8–5.7) while, with redefined cut-offs, the corresponding HRs were 2.7 (95% CI: 1.8–4.0) and 5.8 (95% CI: 4.0–8.3) respectively. Mortality was higher with concurrent pulmonary hypertension (HR 2.0; 95% CI: 1.5–2.9) and lung cancer (HR 2.6; 95% CI: 1.3–4.9).

**Conclusions:**

EMPIRE, one of the largest long-term registries of patients with IPF, provides a more accurate confirmation of prognostic factors and co-morbidities on longer term five-year mortality. It also suggests that some fine-tuning of the indices for mortality may provide a more accurate long-term prognostic profile for these patients.

## Background

Idiopathic pulmonary fibrosis (IPF) is a specific form of chronic fibrosing interstitial pneumonia of unknown cause, occurring primarily in older adults [1]. IPF is a severe disease characterised by progressive worsening in lung function and associated with poor prognosis. While IPF is ultimately fatal, with an estimated median survival of 2–5 years, its clinical course is variable with a rapid decline in lung function in some patients and slower progression in others [2, 3]. Although it is generally assumed that IPF is a rare disease, more recent studies suggest that the incidence might be higher than previously thought [4].

In recent years, two anti-fibrotic drugs - pirfenidone and nintedanib - have been approved for the treatment of IPF in Europe based on large randomised clinical trials [5–9]. Registries of patients with IPF with extended follow-up are necessary to evaluate the effectiveness and safety of these IPF treatments in the real-world setting [10, 11]. Multicentre IPF registries, important to collect such data and describe the epidemiology, the natural course and the clinical management of IPF, have already been implemented in Germany, the United States, Sweden, Australia, the United Kingdom, and Greece [12–17]. However, the existing IPF registries are relatively small in sample size and restricted to a single country, with only limited data from Central and Eastern Europe [12, 18]. Moreover, the validation of valuable prognostic tools such as the GAP index in other populations has been constrained by the relatively small cohort sizes and short follow-up [19–21].

We use the large European MultiPartner IPF Registry (EMPIRE) to address these limitations. We describe the clinical characteristics of these patients and assess predictors of survival over a long follow-up period spanning over 6 years.

## Methods

### The European MultiPartner IPF registry (EMPIRE)

EMPIRE is a multinational, observational longitudinal registry designed to describe the characteristics and outcomes of patients with IPF in 11 Central and Eastern European countries. A detailed description of the registry is provided in the (Additional file 1: Appendix). Briefly, this registry was initiated in 2014 and has been enrolling patients at 48 sites from ten countries as of October 2018 (Additional file 1: Figure S1). The registry was approved according to national regulations in each participating country and has ethical approval to operate in all participating centres. Enrollment of patients and data collection are in compliance with the ethical principles detailed in the Declaration of Helsinki. To be eligible to participate in the registry, patients have to be at least 18 years of age with a diagnosis of IPF according to the diagnostic criteria of IPF based on the international guidelines [1] as assessed by a face-to-face multidisciplinary discussion. This includes patients with prevalent IPF diagnosed before, and incident patients diagnosed after registry initiation in September 2014. Even though most IPF patients are diagnosed at the age of 45 years or older, the minimum age of 18 years allows inclusion of patients with familial IPF. All participating patients must provide written informed consent. Follow-up visits are every 3 or 6 months, following standard clinical practice at each centre. Patient data are collected by the treating physician in a structured and non-interventional manner. Additional file 1: Table S1, lists a selection of variables collected in EMPIRE. Patients are followed in the registry until death or lung transplantation.

### Study cohort

The study cohort included all patients with IPF enrolled in the EMPIRE registry who had information on baseline pulmonary function on TL_CO_, FVC, and FEV_1_, and on follow-up. Patients were followed from IPF diagnosis until death from any cause, lung transplant or last follow-up visit.

### Statistical analysis

Descriptive statistics were used to summarize the demographic and clinical characteristics of IPF patients at enrollment. For the analysis of survival, the Kaplan–Meier method was used to estimate survival over time from diagnosis until death from any cause. Because the registry includes both patients with newly and previously diagnosed IPF, we accounted for left truncation arising from the delay between diagnosis and enrollment into the registry by placing patients in the risk set only from their time of enrollment. For example, a patient enrolled 1 year after diagnosis and followed up in the registry for 3 years was considered to enter the risk set at 12 months and was right censored at 48 months [22].

The Cox proportional hazards regression model was used to identify independent prognostic factors of survival up to 5 years after IPF diagnosis. Continuous factors, including age, FVC and TL_CO_ were analysed using Cox proportional hazards regression model with restricted cubic splines, using four knots. This model fits a curve using a cubic polynomial function separately within five mutually exclusive intervals, imposing that the curves join at the boundaries to generate a smooth continuous function. Prognostic factors were also classified by categories from the GAP index, namely age at diagnosis as ≤60, 61–65 and > 65 years, FVC as < 50, 50–75, and > 75% of the predicted value, and TL_CO_ as ≤35, 36–55, > 55% of the predicted value [23]. The results of the spline analyses were used to redefine cut-points for prognostic variables that make up the GAP index. Finally, an analysis based on the three stages of the GAP index was performed to compare the mortality of these patients. As the GAP index was designed to predict 3-year mortality, we also assessed survival up to 3 years after IPF diagnosis. All models were adjusted for all other independent prognostic factors in multivariate analyses. Independent prognostic factors included age at diagnosis, sex, predicted FVC, predicted TL_CO_, pulmonary hypertension, lung cancer, and long-term oxygen therapy. Data were analysed using SAS (Version 9.4) and R (version 3.5.0).

## Results

The registry enrolled 2789 patients between 1996 and 2018. We excluded 1169 patients, primarily those who only had a baseline visit (305) and those with no or partial lung function data (650), described in Additional file 1: Table S2. Thus, the final study cohort included 1620 patients with IPF who were enrolled as of October 12, 2018 (Additional file 1: Figure S2). A majority of the patients (74.9%) were enrolled into the registry within 6 months after diagnosis (“incident” subjects), with 25.1% considered “prevalent” subjects at the time of enrollment. The mean time from IPF diagnosis to enrollment was 9.7 months.

Table 1 displays the baseline characteristics of the cohort and separately for the incident and prevalent patients. The mean age at diagnosis was 67.6 years, with most patients male (71.4%) and 62.9% with a history of smoking. The mean duration of symptoms prior to diagnosis was 1.6 years. Pulmonary function tests at enrollment showed a mean predicted FEV_1_ of 86.5%, FVC of 78.7%, TL_CO_ of 46.3%, and a mean 6-min walk distance of 388.2 m. The distribution of GAP stage was I (47.7%), II (43.8%) and III (8.5%). Approximately 40% of all patients were receiving or had received pharmacological treatment for IPF at or prior to enrollment, which included a history of anti-fibrotic medication as well as other immunosuppressive combined regimen, but does not include medications received after enrollment. The incident and prevalent subjects were generally similar.

**Table 1:** Baseline characteristics of IPF patients enrolled into the EMPIRE registry, overall and accoding to time from diagnosis to registry enrollment

	All patients	Time from diagnosis to enrollment < 6 months	Time from diagnosis to enrollment ≥6 months
Characteristics	*n* = 1620	*n* = 1213 (74.9%)	*n* = 407 (25.1%)
Country, n (%)			
Austria	26 (1.6)	10 (0.8)	16 (3.9)
Bulgaria	3 (0.2)	2 (0.2)	1 (0.2)
Croatia	40 (2.5)	32 (2.6)	8 (2.0)
Czech Republic	683 (42.2)	591 (48.7)	92 (22.6)
Hungary	112 (6.9)	89 (7.3)	23 (5.7)
Israel	78 (4.8)	41 (3.4)	37 (9.1)
Poland	255 (15.7)	125 (10.3)	130 (31.9)
Serbia	55 (3.4)	38 (3.1)	17 (4.2)
Slovakia	110 (6.8)	87 (7.2)	23 (5.7)
Turkey	258 (15.9)	198 (16.3)	60 (14.7)
Time from diagnosis to enrollment [years], mean (SD)	0.8 (2.0)	0.1 (0.1)	3.1 (3.0)
Duration of symptoms prior to diagnosis [years], mean (SD)	1.6 (2.1)	1.6 (1.8)	1.5 (2.7)
Age at diagnosis [years], mean (SD)	67.6 (8.9)	68.4 (8.6)	65.4 (9.5)
Male sex, n (%)	1157 (71.4)	878 (72.4)	279 (68.6)
BMI [kg/m2], mean (SD)	28.4 (4.4)	28.5 (4.4)	28.1 (4.5)
History of smoking, n (%)	1019 (62.9)	767 (63.2)	252 (61.9)
Familial IPF, n (%)	55 (3.4)	44 (3.6)	11 (2.7)
Diagnosis based on, n (%)			
Clinical signs	1286 (79.4)	988 (81.5)	298 (73.2)
Radiological patterns	1590 (98.2)	1194 (98.4)	396 (97.3)
Histopathological patterns	349 (21.5)	261 (21.5)	88 (21.6)
FEV_1_ [% predicted], mean (SD) ^a^	86.5 (17.2)	86.4 (16.8)	87.1 (18.2)
FVC [% predicted], mean (SD) ^a^	78.7 (19.7)	77.3 (19.4)	82.7 (19.7)
TL_CO_ [% predicted], mean (SD) ^a^	46.3 (20.9)	46.6 (20.6)	45.5 (21.6)
6MWD [m], mean (SD) ^a^	388.2 (111.0)	387.7 (109.2)	389.6 (115.8)
GAP stage, n (%)			
I	772 (47.7)	536 (44.2)	236 (58.0)
II	710 (43.8)	565 (46.6)	145 (35.6)
III	138 (8.5)	112 (9.2)	26 (6.4)
Treatment (current or past), n (%)			
Pharmacological	618 (38.1)	410 (33.8)	208 (51.1)
Clinical trial	111 (6.9)	53 (4.4)	58 (14.3)
Rehabilitation	427 (26.4)	352 (29.0)	75 (18.4)
LTOT	327 (20.2)	218 (18.0)	109 (26.8)

*Abbreviations: 6MWD* 6-min walk distance, *BMI* body mass index, *FEV*_*1*_ forced expiratory volume, *FVC* forced vital capacity, *IPF* idiopathic pulmonary fibrosis, *LTOT* long-term oxygen therapy, *SD* standard deviation, *TL*_*CO*_ carbon monoxide transfer factor

^a^If measurement was not available at enrollment, data measured at diagnosis were used

The mean and median follow-up from enrollment were 1.6 and 1.3 (maximum 6.7) years respectively. There were 357 deaths during follow-up. One-year survival from diagnosis was 89.2%, while it was 65.5 and 46.4% at 3 and 5 years, respectively, with a median survival of 4.5 years.

Table 2 presents the adjusted hazard ratios (HR) of mortality from the time of IPF diagnosis for the significant independent predictors of this outcome. In addition to the known factors in the GAP stage (sex, age, FVC and TL_CO_), the presence of pulmonary hypertension (HR 2.04; 95% CI: 1.46–2.86), lung cancer (HR 2.55; 95% CI: 1.33–4.92), and long-term oxygen therapy (HR 1.49; 95% CI: 1.19–1.88) at the time of diagnosis were all found to increase the risk of death during follow-up.

**Table 2:** Crude and adjusted hazard ratios (HR) of 5-year mortality from time since IPF diagnosis, for the significant baseline predictors at diagnosis, with categories defined by the GAP index, as estimated by the Cox proportional hazards model

	Number (%)	Crude HR	Adjusted HR^a^ (95% CI)
Age at diagnosis			
> 65 years	1018 (63)	1.25	1.52 (1.14–2.03)
61–65 years	274 (17)	0.99	1.01 (0.70–1.47)
≤ 60 years	328 (20)	Reference	Reference
Male sex	1157 (71)	1.76	1.51 (1.15–1.96)
FVC (% predicted)			
< 50%	78 (5)	5.62	3.45 (2.28–5.23)
50–75%	669 (41)	2.66	1.89 (1.47–2.42)
> 75%	873 (54)	Reference	Reference
TL_CO_ (% predicted)			
≤ 35%	419 (26)	6.22	3.52 (2.40–5.18)
36–55%	687 (42)	3.90	3.05 (2.13–4.36)
> 55%	514 (32)	Reference	Reference
Pulmonary hypertension	123 (8)	2.63	2.04 (1.46–2.86)
Lung cancer	19 (1)	3.47	2.55 (1.33–4.92)
Long-term oxygen therapy	327 (20)	2.35	1.49 (1.19–1.88)

*Abbreviations: FVC* forced vital capacity, *TL*_*CO*_ carbon monoxide transfer factor

^a^Adjusted for one another, concordance index = 0.76

**Fig. 1:**
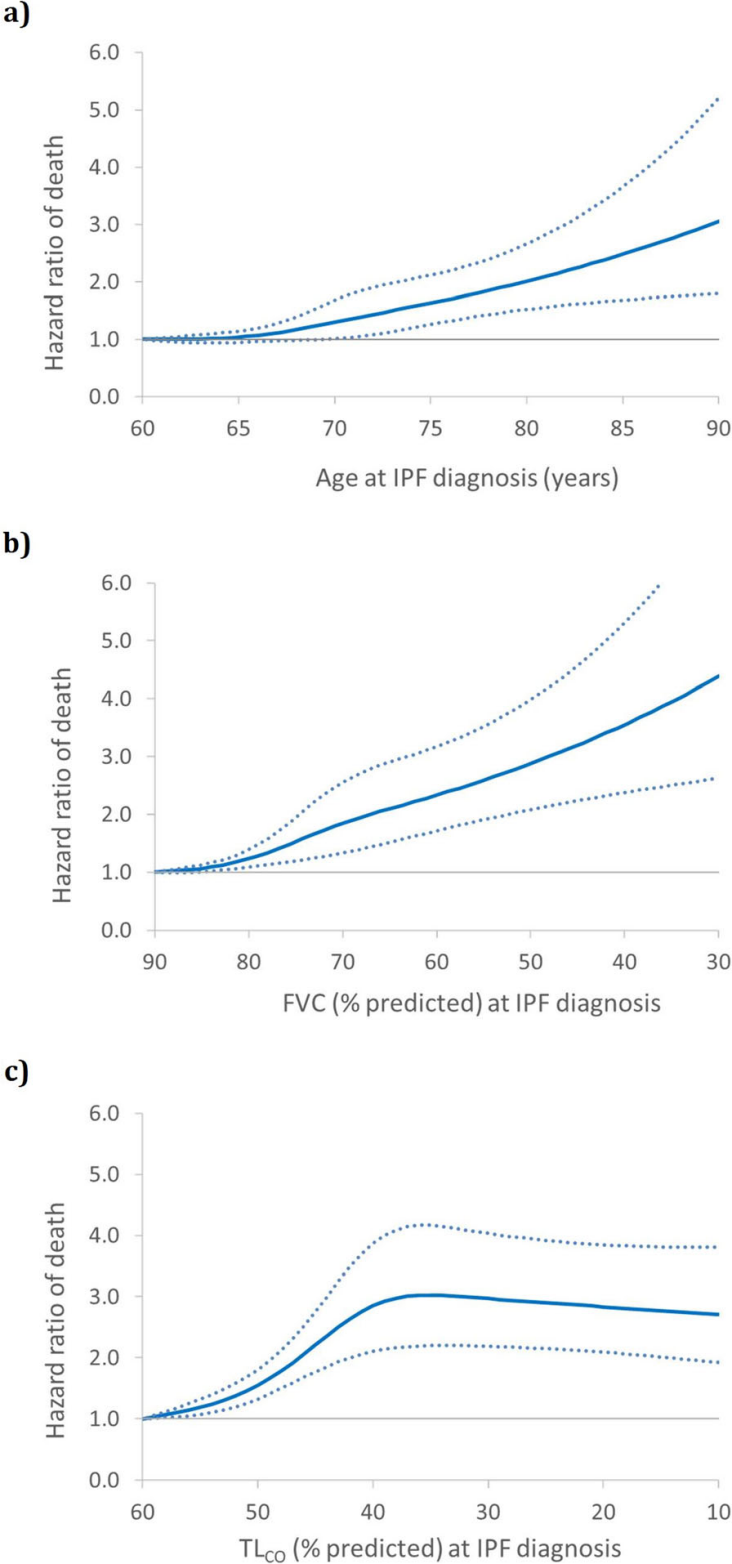
Smooth cubic spline curve of the adjusted hazard ratio (solid line) and 95% CIs (dotted lines) of death, estimated by Cox proportional hazards model, as a function of: **a**) age at IPF diagnosis; **b**) % predicted FVC; and **c**) % predicted TL_CO_

Figure 1 displays the mortality fitted by cubic splines for the continuous forms of age, FVC and TL_CO_. For age at diagnosis, the increase in mortality starts at 65 years and is gradual until 90 (Fig. 1a). For FVC, the increase starts when FVC is 85% of predicted and increases gradually with decreasing FVC, reaching a four-fold increase when FVC reaches 30% of predicted (Fig. 1b). For TL_CO_, the increase starts gradually at 60% predicted, reaching a plateau of a 3-fold increase at 40% predicted, remaining constant up to 10% predicted (Fig. 1c). These analyses suggest a readjustment of some cut-points for the prognostic variables that make up the GAP index, namely age at diagnosis as ≤65, 66–74 and > 75 years, and TL_CO_ as ≤55 and > 55% of the predicted value (Table 3).

**Table 3:** Crude and adjusted hazard ratios (HR) of 5-year mortality from time since IPF diagnosis, for the significant baseline predictors at diagnosis, with redefined cut-points for age and TL_CO_ categories defining the GAP index^b^, estimated by Cox proportional hazards model

	n (%)	Crude HR	Adjusted HR^a^ (95% CI)
Age at diagnosis			
≥ 75 years	353 (22)	1.54	2.16 (1.61–2.89)
65–74 years	730 (45)	1.16	1.34 (1.04–1.74)
< 65 years	537 (33)	Reference	Reference
Men	1157 (71)	1.76	1.52 (1.16–1.97)
Predicted FVC			
< 50%	78 (5)	5.62	3.80 (2.53–5.70)
50–75%	669 (41)	2.66	1.98 (1.55–2.54)
> 75%	873 (54)	Reference	Reference
Predicted TL_CO_			
≤ 55%	1106 (68)	4.63	3.22 (2.27–4.56)
> 55%	514 (32)	Reference	Reference

^a^Adjusted for all other predictors, concordance-index = 0.75

^b^ Scores based on magnitude of hazard ratios

For the GAP index, the crude hazard ratio of mortality for stage II is 3.32 (95% CI: 2.59–4.26) and for stage III it is 5.12 (95% CI: 3.60–7.28), compared to stage I (Table 4). When adjusted for pulmonary hypertension, lung cancer and long-term oxygen therapy, the hazard ratio of mortality for stage II is 2.89 (95% CI: 2.25–3.73) and for stage III it is 3.97 (95% CI: 2.76–5.71), compared to stage I. The results for 3-year mortality were similar (Additional file 1: Table S3). When the cut-offs were readjusted, along with weights proportional to the hazard ratios, the corresponding adjusted hazard ratio of mortality for stage II is 2.66 (95% CI: 1.80–3.94) and for stage III it is 5.79 (95% CI: 4.03–8.31), compared to stage I (Table 4).

**Table 4:** Crude and adjusted hazard ratios (HR) of 5-year mortality from time since IPF diagnosis for the original GAP stages and with redefined cut-points, as estimated by the Cox proportional hazards model

	Number (%)	Crude HR (95% CI)	Adjusted HR^a^ (95% CI)
GAP stage			
I	772 (47.7)	Reference	Reference
II	710 (43.8)	3.32 (2.59–4.26)	2.89 (2.25–3.73)
III	138 (8.5)	5.12 (3.60–7.28)	3.97 (2.76–5.71)
GAP stage with redefined cut-points			
I	488 (30.1)	Reference	Reference
II	485 (29.9)	3.22 (1.97–4.31)	2.66 (1.80–3.94)
III	647 (39.9)	6.92 (4.86–9.85)	5.79 (4.03–8.31)

^a^Adjusted for pulmonary hypertension, lung cancer and long-term oxygen therapy, concordance index = 0.81 and 0.77 respectively

**Fig. 2:**
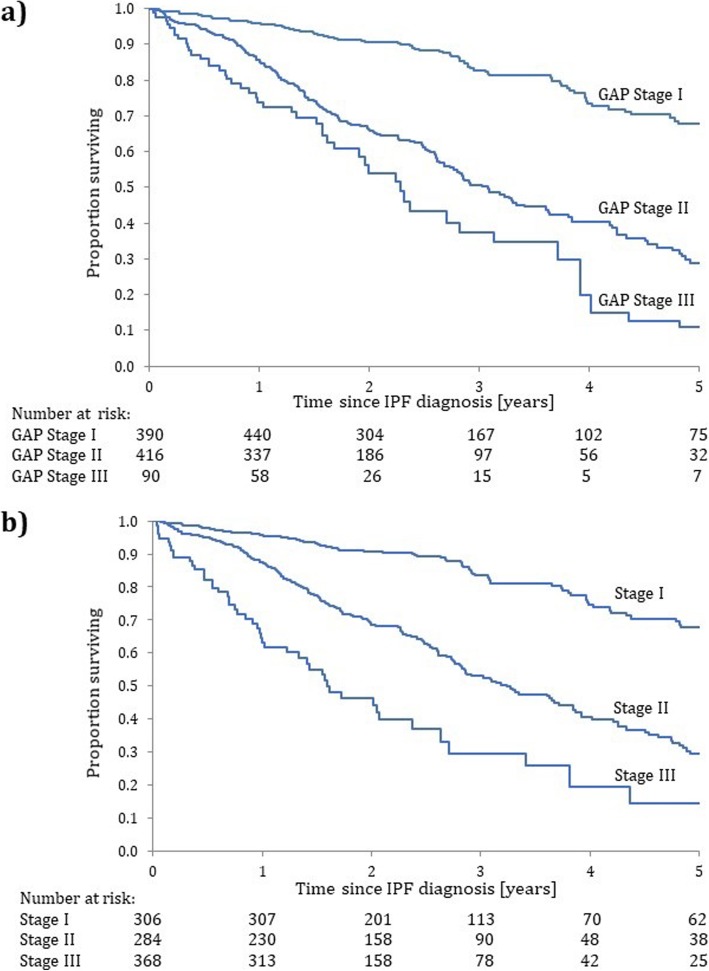
Kaplan–Meier survival functions for the cohort of 1620 patients with IPF from the time of their IPF diagnosis over a 5-year follow-up period, according to the **a**) original GAP stage classification and **b**) with redefined cut-points

The Kaplan-Meier survival curves according to the standard GAP stage classification show clear separations (Fig. 2a), though these are somewhat more distinct with the redefined cut-points, i.e. the survival curve for stage II rises (Fig. 2b).

## Discussion

Using a cohort of over 1600 patients with IPF from the EMPIRE registry, its long-term follow-up allowed for accurate estimates of mortality after diagnosis, finding that only 46% of patients survive to 5 years after diagnosis. Even though the GAP index was designed to predict mortality in the first 3 years after diagnosis, this study confirms the prognostic value of the GAP stages on mortality at 5 years post-diagnosis. It also suggests that some minor redefinition of the categories of age and TL_CO_ could improve the prognostic accuracy of the GAP index. We also found that pulmonary hypertension, lung cancer and long-term oxygen were associated with higher mortality, above and beyond the effect of GAP.

IPF registries have been implemented in Germany, the United States, Sweden, Australia, the United Kingdom, and Greece [12–17]. However, large registries based on multiple countries are scarce. EMPIRE includes complete data on over 1600 patients from ten countries in Central and Eastern Europe. Patients enrolled in the EMPIRE registry have similar patient characteristics to those reported in the previous literature [24–27]. The EMPIRE registry data confirm a more favourable prognosis after IPF diagnosis for female sex, younger age, greater predicted FVC, and greater predicted TL_CO_ at diagnosis, all factors of the GAP index. Additionally, IPF patients with pulmonary hypertension, lung cancer, or those on long-term oxygen therapy have poorer prognosis. These findings are consistent with the data reported in previous studies, although the higher mortality with pulmonary hypertension was not shown in all other studies [2, 28–31]. Indeed, the recent study by Kreuter et al. was based on 272 subjects and found that PH was not independently associated with mortality (HR 1.1; CI 0.7–1.7), though the upper confidence limit cannot rule out a HR of 1.7, while our much larger study found a HR of 2.0 (CI 1.5–2.9). Pulmonary hypertension and lung cancer add to the symptoms and decline in pulmonary function, and other physiological consequences of IPF which can explain the increased risk of death among IPF patients with these co-morbidities [32]. The association between long-term oxygen therapy and mortality likely characterizes severity of disease [33].

Previous studies reporting survival according to the GAP staging present only crude analyses [34, 35]. When we adjusted the effects from the GAP stages for pulmonary co-morbidity, we noted that effect of the stage III group on mortality was reduced compared with stage I. This corroborates the recent trend to introduce co-morbidity in prognostic indices [36]. Our cubic spline analyses of the continuous predictors age at diagnosis, FVC, and TL_CO_ also showed that the GAP categories may merit a reassessment in future indices. For example, we found no difference in mortality (HR 1.01; 95% CI: 0.70–1.47) between patients < 60 years of age (score = 0 in GAP) and those 61–65 years (score = 1). We also found that the effect on mortality was of the same magnitude with TL_CO_ ≤ 35% (score = 2) and 36–55% (score = 1) predicted, which has not been reported before. When these were redefined, the corresponding adjusted hazard ratio of mortality for stages II and III were 2.7 and 5.8 respectively, while for the standard stages II and III it was 2.9 and 4.0, compared to stage I. These results suggest that it may be useful to re-examine the commonly used thresholds in large cohorts of patients with IPF, while incorporating pulmonary co-morbidity.

This study has several strengths, including using data from the EMPIRE multinational IPF registry which enrols a large, well-defined and diverse IPF patient population from Central and Eastern Europe, with sufficient numbers in relevant subgroups such as age < 60 years or TL_CO_ ≤ 35% predicted. Our study included 419 (26%) patients with TL_CO_ ≤ 35% predicted, which likely provides a more accurate representation of the association with mortality in this group, compared to smaller studies. Indeed, the largest study providing mortality data only had 94 patients with TL_CO_ ≤ 35% predicted [37]. Also, the long follow-up until death allowed to identify predictors of long-term survival after IPF diagnosis. A major strength of the registry is the large proportion of patients (75%) who were included within 6 months of diagnosis, thus avoiding potential biases due to prevalent cohorts. Finally, the spline analysis of the continuous predictors allowed a more accurate understanding of these prognostic predictors of mortality, and to verify the assumptions of linear relationships made by other studies.

Limitations include differences with patients who chose not to participate, a limiting factor regarding generalizability. Approximately 60% of the patients did not have pharmacological treatment reported at enrollment, though this included patients diagnosed at enrollment and thus might not have had an IPF-related medication history yet. Finally, the exclusion of patients with missing data on pulmonary function and follow-up decreased the size of the study cohort, though the cohort of 1620 patients was still larger than in most studies in patients with IPF. Overall, excluded patients were similar to patients in the study cohort. While the 302 patients with missing lung function data had generally similar patient characteristics, the 348 patients with incomplete lung function data were somewhat more likely on long-term oxygen therapy and seemed to have, slightly lower predicted lung function measures, such as TL_CO_ and FVC. However, these values were based on a limited subset of patients with partial information and need to be interpreted with caution. The 305 patients excluded because of only one baseline visit were generally similar to patients included in the study cohort. These were mainly patients recently enrolled in the registry in 2018 and thus had not yet had another follow-up visit yet. Therefore, the exclusion of patients with incomplete data in this study could have led to decreased precision due to a smaller sample size but unlikely to have introduced selection bias.

Future studies evaluating IPF therapies need to consider confounding by indication and potential prevalent user biases as registries typically enrol prevalent and incident patients. This incident-prevalent phenomenon can lead to unexpected findings such as zero deaths in the first 6–12 months of follow-up [38]. The EMPIRE registry avoided the survival bias from incident-prevalent cohorts by enrolling about 75% of patients within 6 months of diagnosis and using the proper left truncation survival analyses [39].

## Conclusions

The current enrollment of more than 2500 IPF patients in the EMPIRE registry since September 2014 is a promising cohort size for future clinical research in IPF, making it one of the largest ongoing multinational registries. Data from this registry will provide valuable longitudinal real-world data to describe regional characteristics of patients with IPF, including co-morbidities and complications of IPF, the quality of life of patients with IPF and management of the disease.

In all, with its large size of over 1600 patients with IPF with long follow-up, the EMPIRE registry provided some novelty by its more accurate confirmation of the prognostic factors and co-morbidities on longer term five-year mortality. Its results suggest that some fine-tuning of the commonly used classification indices of mortality may be possible to provide a more accurate long-term prognostic profile for these patients.

## Supplementary information

**Additional file 1:** EMPIRE. Supplementary information: Detailed description of EMPIRE, supplementary Figures and Tables.

## Data Availability

The dataset analysed during the current study are available from the corresponding author on reasonable request.

## Abbreviations

CI: Confidence interval
EMPIRE: European Multipartner IPF Registry
FEV_1_: Forced expiratory volume
FVC: Forced vital capacity
HR: Hazard ratio
IPF: Idiopathic pulmonary fibrosis
TL_co_: Carbon monoxide transfer factor
