# Supplementary material for: The European MultiPartner IPF registry (EMPIRE): validating long-term prognostic factors in idiopathic pulmonary fibrosis
Source: Respir Res. 2020 Jan 8;21:11. doi: 10.1186/s12931-019-1271-z (PMC6951015; doi:10.1186/s12931-019-1271-z)
Supplement: Supplementary file 1 — Additional file 1: EMPIRE. Supplementary information: Detailed description of EMPIRE, supplementary Figures and Tables. [file 12931_2019_1271_MOESM1_ESM.docx]

**The European MultiPartner IPF Registry (EMPIRE): Validating long-term prognostic factors in idiopathic pulmonary fibrosis**

**Additional file 1**

**METHODS: Description of the European MultiPartner IPF Registry (EMPIRE)**

**Study Design**

EMPIRE is a multinational, prospective, observational longitudinal registry designed to describe the characteristics and management of newly diagnosed and prevalent patients with IPF in Central and Eastern Europe. The registry was initiated in the Czech Republic in September 2014 and is based on the Czech IPF registry (1) which was started in 2012. As of October 2018, the registry included 48 sites from ten countries (e-Figure 1), namely Austria (1 site), Bulgaria (1 site), Croatia (3 sites), Czech Republic (17 sites), Hungary (6 sites), Israel (1 site), Poland (9 sites), Serbia (3 sites), Slovakia (6 sites), Turkey (1 site). Additional sites have been enrolled in Macedonia since then. Patients are enrolled at participating centres based on the diagnoses and assessment of the treating physician. Participating physicians are not subject to any instructions with regard to the diagnosis and therapy of their patients. All examinations performed depend on the discretion and clinical routine of the treating physician. Follow-up visits are every 3 or 6 months, following standard clinical practice at each centre. Patients are followed until death or lung transplantation. Currently, the end of enrollment is still undefined.

The steering committee of the EMPIRE registry consists of representatives from all participating countries (Austria, Bulgaria, Czech Republic, Croatia, Hungary, Israel, Macedonia, Poland, Serbia, Slovakia, Turkey) and has the overall responsibility for the conduct of the study according to Good Clinical Practice as well as all applicable requirements imposed by the reviewing Ethics Committee. The registry was approved according to national regulations in each participating country. The registry has ethical approval to operate in all participating centres.

**Patients**

To be eligible to participate in the registry, patients have to be at least 18 years of age with a diagnosis of IPF according to the diagnostic criteria of IPF based on the 2011 international guidelines by the European Respiratory Society, the American Thoracic Society, the Japanese Respiratory Society, and the Latin American Thoracic Association (2) as assessed by the treating physician. Since the establishment of the EMPIRE registry, diagnostic guidelines for IPF have been updated in 2018 (3). As the updated diagnostic criteria are less strict compared to the 2011 criteria, all current included patients should also fulfill the new diagnostic criteria for IPF. This includes patients with prevalent disease diagnosed before, and incident patients diagnosed after registry initiation in September 2014. Even though most IPF patients are diagnosed at the age of 45 years or older, the minimum age of 18 years allows inclusion of patients with familial IPF. Enrollment of patients and data collection are in compliance with the ethical principles detailed in the Declaration of Helsinki. All participating patients must provide confirmed consent prior to enrollment. Patients are enrolled in a consecutive manner at each site. Participating patients are treated according to standard clinical practice of each centre.

**Data collection and management**

Patient data are collected by the treating physician. The frequency of the follow-up visits is at the discretion of the treating physician which is usually every 3 to 6 months. All data are collected in a structured and non-interventional manner until the patient’s death or loss to follow-up. Supplementary Table 1 lists variables collected in EMPIRE. At baseline, i.e. the time of enrollment into the registry, all available clinical data from the first symptoms prior to enrollment are collected. Data include information on demographics, diagnostic evaluation of IPF, IPF symptoms, potential IPF risk factors, comorbidities, previous non IPF-related medications, and current IPF disease management. The following data are collected at each follow-up visit every 3 to 6 months after enrollment: changes in current disease status, acute exacerbations, IPF symptoms, functional assessment (lung function tests, 6-minute walk test), physician assessment, IPF disease management and treatment, resource utilization (hospitalisations, IPF-related physician visits), treatment outcomes, adverse events, comorbidities, non-IPF related medications, and quality of life which will be assessed using the EuroQoL or the recently introduced King´s brief interstitial lung disease questionnaire (4, 5). Information on reasons for the end of follow-up and on the cause of death are also collected.

Data on IPF treatment include pharmacological treatments, participation in clinical trials, rehabilitation, long-term oxygen therapy, and lung transplantation. Disease outcomes include the decrease in forced vital capacity (FVC) or forced expiratory volume (FEV_1_), decrease in the carbon monoxide transfer factor (TL_CO_), lung transplantation, and death.

Data of the EMPIRE project are stored in an online and highly secure database system. Data entry is performed using electronic case report forms by qualified personnel. Privacy of patients is protected by de-identified data collection using patient unique identifiers. All electronic case report form pages are subject to initial inspection for omitted data, gross data inconsistencies, illegible data and deviations by consultants before statistical analysis. Intermittent data reviews, including crosschecks, are performed and any discovered inconsistencies are reported to the study site and corrected if necessary. Centralised administration and uniform forms for data collection at all sites facilitate synthesis of the data and comparability between sites.

**References:**

1. Doubkova M, Uher M, Bartos V, Sterclova M, Lacina L, Lostakova V, et al. [Idiopathic pulmonary fibrosis prognostic factors - analysis of the Czech registry]. Cas Lek Cesk. 2016;155(4):22-8.

2. Raghu G, Collard HR, Egan JJ, Martinez FJ, Behr J, Brown KK, et al. An official ATS/ERS/JRS/ALAT statement: idiopathic pulmonary fibrosis: evidence-based guidelines for diagnosis and management. Am J Respir Crit Care Med. 2011;183(6):788-824.

3. Raghu G, Remy-Jardin M, Myers JL, Richeldi L, Ryerson CJ, Lederer DJ, et al. Diagnosis of Idiopathic Pulmonary Fibrosis. An Official ATS/ERS/JRS/ALAT Clinical Practice Guideline. Am J Respir Crit Care Med. 2018;198(5):e44-e68.

4. EuroQol--a new facility for the measurement of health-related quality of life. Health Policy. 1990;16(3):199-208.

5. Patel AS, Siegert RJ, Brignall K, Gordon P, Steer S, Desai SR, et al. The development and validation of the King's Brief Interstitial Lung Disease (K-BILD) health status questionnaire. Thorax. 2012;67(9):804-10.

**Supplementary Figure S1.** Countries in Central and Eastern Europe participating in the European MultiPartner IPF Registry (EMPIRE).


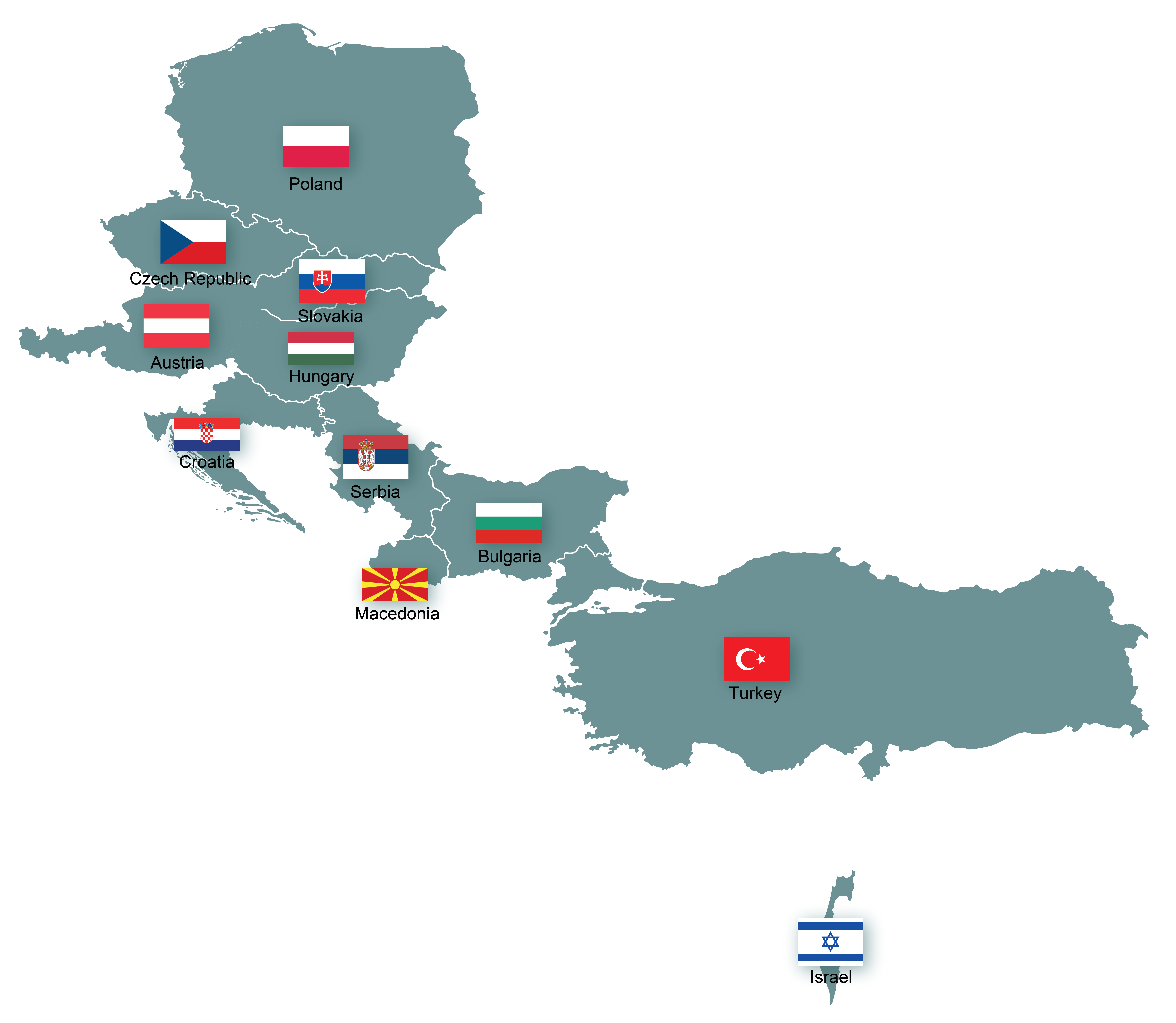

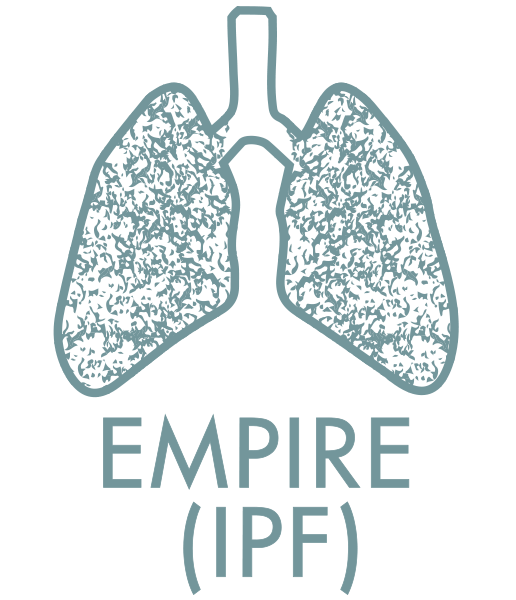


**Supplementary Figure S2.** Flowchart of formation of the study cohort.

EMPIRE registry

(initiated in 2012 (Czech Republic), multi-national in 2014)

2789 IPF patients

First visit: Dec 20, 1996 – Oct 12, 2018

2575 IPF patients

44 - No date of diagnosis

15 - No date of first visit

141 - Enrolled before 2012

3 - Age <18 years or 0

11 – Missing date of death

305 – Only baseline visit

2273 patients with some pulmonary function data at enrollment or diagnosis

(FVC, FEV1 or TL_CO_)

302 - No information on pulmonary function

1925 patients with complete pulmonary function data at enrollment or diagnosis

(FVC, FEV1, TL_CO_)

**1620 patients with complete lung function data at enrollment or diagnosis**

**(FVC, FEV1, TL_CO_)**

n(incident) = 1213 (74.9%)

n(prevalent) = 407 (25.1%)

**Supplementary Table S1.** Variables collected (if available) in the European MultiPartner IPF Registry.

| **Time of data collection** | **Content** | |
| --- | --- | --- |
| **At enrolment** | **Demographic information** | Age at enrolment |
|  |  | Date of birth |
|  |  | Sex |
|  |  | Race |
|  |  | Date of visit (date of enrolment) |
|  |  | Centre |
|  |  | Height |
|  |  | Weight |
|  | **IPF diagnosis** | Date of diagnosis |
|  |  | Age at diagnosis |
|  |  | Duration of symptoms prior to diagnosis |
|  |  | Familial IPF |
|  |  | Base for diagnosis  - Clinical signs  - Radiological patterns  - Histopathological patterns |
|  | **(Potential) IPF risk factors** | Family history |
|  |  | Cigarette smoking |
|  |  | Occupational/environmental exposure |
|  |  | Medication/treatment exposures |
|  | **IPF symptoms** | Dyspnoea |
|  |  | Finger clubbing |
|  |  | Crepitus on auscultation |
| **At enrolment and follow-up,**  **every 3 or 6 months** | **Physician assessment** | Evaluation of current IPF status |
|  | **Radiology** | HRCT |
|  | **Pathology** | Bronchoalveolar lavage |
|  |  | Lung biopsy |
|  | **Lung function** | Pulmonary function tests (FEV_1_, FVC, TL_CO_) |
|  |  | 6-minute walk test |
|  |  | Spiro-ergometry |
|  | **IPF treatment** | Medication |
|  |  | Participation in IPF trial |
|  |  | Rehabilitation |
|  |  | Long-term oxygen therapy |
|  | **Adverse events** |  |
|  | **Non-IPF medication** |  |
|  | **Comorbidities** |  |
| **At follow-up** | **Quality of Life** | EuroQoL questionnaire |
|  | **Resource utilization** | Number of physician contacts since enrolment |
|  |  | Hospitalisation |
|  | **End of observation** | Death |
|  |  | Lung transplantation |
|  |  | Reason for loss to follow-up |

Abbreviations: FEV_1_, forced expiratory volume; FVC, forced vital capacity; HRCT, high-resolution computed tomography; IPF, idiopathic pulmonary fibrosis; TL_CO_, carbon monoxide transfer factor.

**Supplementary Table S2.** Baseline characteristics of patients with IPF excluded from the study cohort.

|  | All excluded patients | Patients with partial information on lung function | Patients with no information on lung function | Patients with no FU, all lung function available |
| --- | --- | --- | --- | --- |
| **Characteristics** | n =955 | n = 348 | n = 302 | n=305 |
| Country, n (%) |  |  |  |  |
| Austria | 33 (3.5) | 7 (2.0) | 3 (1.0) | 26 (1.6) |
| Bulgaria | 8 (0.8) | 0 | 1 (0.3) | 7 (2.3) |
| Croatia | 39 (4.1) | 17 (4.9) | 2 (0.7) | 20 (6.6) |
| Czech Republic | 155 (16.2) | 48 (13.8) | 64 (21.2) | 43 (14.1) |
| Hungary | 120 (12.6) | 49 (14.1) | 43 (14.2) | 28 (9.2) |
| Israel | 51 (5.3) | 33 (9.5) | 6 (2.0) | 12 (3.9) |
| Poland | 195 (20.4) | 52(14.9) | 43 (14.2) | 100 (32.8) |
| Serbia | 33 (3.5) | 14 (4.0) | 14 (4.6) | 5 (1.6) |
| Slovakia | 69 (7.2) | 15 (4.3) | 41 (13.6) | 13 (4.3) |
| Turkey | 252 (26.4) | 113 (32.5) | 85 (28.1) | 54 (17.7) |
| Time from diagnosis to enrollment [years], mean (SD) | 1.6 (2.7) | 1.9 (3.1) | 1.9 (2.7) | 0.9 (1.9) |
| Duration of symptoms prior to diagnosis [years], mean (SD) | 1.4 (2.0) | 1.5 (2.3) | 1.3 (1.5) | 1.5 (2.1) |
| Age at diagnosis [years], mean (SD) | 66.2 (9.7) | 64.6 (10.3) | 66.2 (9.3) | 68.1 (9.3) |
| Male sex, n (%) | 638 (66.8) | 235 (67.5) | 207 (68.5) | 196 (64.3) |
| BMI [kg/m2], mean (SD) | 28.2 (4.4) | 28.0 (4.4) | 28.4 (4.6) | 28.1 (4.2) |
| History of smoking, n (%) | 543 (56.9) | 193 (55.5) | 148 (49.0) | 202 (66.2) |
| Familial IPF, n (%) | 34 (3.6) | 12 (3.4) | 13 (4.3) | 9 (3.0) |
| Diagnosis based on, n (%) |  |  |  |  |
| Clinical signs | 727 (76.1) | 240 (69.0) | 211 (69.9) | 276 (90.5) |
| Radiological patterns | 926 (97.0) | 335 (96.3) | 289 (95.7) | 302 (99.0) |
| Histopathological patterns | 157 (16.4) | 71 (20.4) | 53 (17.5) | 33 (10.8) |
| FEV1 [% predicted], mean (SD) * | 89.0 (18.4) | 85.0 (14.3)** | n/a | 91.2 (19.0) |
| FVC [% predicted], mean (SD) * | 73.8 (26.1) | 57.7 (20.3)** | n/a | 86.2 (23.1) |
| TLCO [% predicted], mean (SD) * | 43.0 (22.0) | 35.5 (18.4)** | n/a | 47.2 (22.8) |
| 6MWD [m], mean (SD) * | 378.2 (121.2) | 362.0 (124.8) | 354.9 (116.9) | 399.6 (115.7) |
| Treatment (current or past), n (%) |  |  |  |  |
| Pharmacological | 356 (37.3) | 140 (40.2) | 127 (42.1) | 89 (29.2) |
| Clinical trial | 54 (5.7) | 27 (7.8) | 19 (6.3) | 8 (2.6) |
| Rehabilitation | 108 (11.3) | 51 (14.7) | 31 (10.3) | 26 (8.5) |
| LTOT | 211 (22.1) | 109 (31.3) | 67 (22.2) | 35 (11.5) |
| * If measurement was not available at enrollment, data measured at diagnosis were used.  ** Computed on less than 348 patients because of partially available data | | | | |

**Supplementary Table S3.** Crude and adjusted hazard ratios (HR) of 3-year mortality from time since IPF diagnosis for a) significant baseline predictors at diagnosis and b) GAP stages, as estimated by the Cox proportional hazards model.

a)

|  | **Number (%)** | **Crude HR** | **Adjusted HR* (95% CI)** |
| --- | --- | --- | --- |
| **Age at diagnosis** |  |  |  |
| > 65 years | 1018 (63) | 1.43 | 1.77 (1.27 - 2.47) |
| 61-65 years | 274 (17) | 1.12 | 1.22 (0.80-1.86) |
| ≤ 60 years | 328 (20) | Reference | Reference |
| **Male sex** | 1157 (71) | 1.76 | 1.48 (1.01 - 1.98) |
| **FVC (% predicted)** |  |  |  |
| <50% | 78 (5) | 5.78 | 3.79 (2.43 - 5.02) |
| 50-75% | 669 (41) | 2.70 | 1.95 (1.49 - 2.56) |
| >75% | 873 (54) | Reference | Reference |
| **TL_CO_ (% predicted)** |  |  |  |
| ≤35% | 419 (26) | 6.49 | 3.62 (2.37 - 5.53) |
| 36-55% | 687 (42) | 4.01 | 3.10 (2.08 - 4.61) |
| >55% | 514 (32) | Reference | Reference |
| **Pulmonary hypertension** | 123 (8) | 2.68 | 2.13 (1.47 – 3.08) |
| **Lung cancer** | 19 (1) | 4.11 | 2.91 (1.51 - 5.63) |
| **Long-term oxygen therapy** | 327 (20) | 2.33 | 1.46 (1.13 - 1.88) |

*****Adjusted for one another, concordance index=0.72

Abbreviations: FVC, forced vital capacity; TL_CO_, carbon monoxide transfer factor.

b)

|  | **Number (%)** | **Crude HR (95% CI)** | **Adjusted HR* (95% CI)** |
| --- | --- | --- | --- |
| **GAP stage** |  |  |  |
| I | 772 (47.7) | Reference | Reference |
| II | 710 (43.8) | 3.65 (2.75 - 4.85) | 3.21 (2.41 - 4.89) |
| III | 138 (8.5) | 5.49 (3.72 - 8.10) | 4.34 (2.90 - 6.49) |

*****Adjusted for pulmonary hypertension, lung cancer and long-term oxygen therapy, concordance index=0.77
